# Method for the determination of natural ester-type gum bases used as food additives via direct analysis of their constituent wax esters using high-temperature GC/MS

**DOI:** 10.1002/fsn3.117

**Published:** 2014-05-07

**Authors:** Atsuko Tada, Kyoko Ishizuki, Takeshi Yamazaki, Naoki Sugimoto, Hiroshi Akiyama

**Affiliations:** 1 National Institute of Health SciencesTokyo, Japan; 2 Jissen Women's UniversityHino, Japan

**Keywords:** Food additive, gum base, high-temperature GC/MS, wax ester

## Abstract

Natural ester-type gum bases, which are used worldwide as food additives, mainly consist of wax esters composed of long-chain fatty acids and long-chain fatty alcohols. There are many varieties of ester-type gum bases, and thus a useful method for their discrimination is needed in order to establish official specifications and manage their quality control. Herein is reported a rapid and simple method for the analysis of different ester-type gum bases used as food additives by high-temperature gas chromatography/mass spectrometry (GC/MS). With this method, the constituent wax esters in ester-type gum bases can be detected without hydrolysis and derivatization. The method was applied to the determination of 10 types of gum bases, including beeswax, carnauba wax, lanolin, and jojoba wax, and it was demonstrated that the gum bases derived from identical origins have specific and characteristic total ion chromatogram (TIC) patterns and ester compositions. Food additive gum bases were thus distinguished from one another based on their TIC patterns and then more clearly discriminated using simultaneous monitoring of the fragment ions corresponding to the fatty acid moieties of the individual molecular species of the wax esters. This direct high-temperature GC/MS method was shown to be very useful for the rapid and simple discrimination of varieties of ester-type gum bases used as food additives.

## Introduction

Natural ester-type gum bases are used worldwide as food additives to provide, for example, specific textures to chewing gums and surface coatings for chocolates and fruits. There are various kinds of natural ester-type gum bases, and each is comprised of a characteristic mixture of wax esters composed of a specific combination of long-chain fatty acids and long-chain fatty alcohols, and thus they each have different properties with respect to their elasticity and flexibility. To assess the safety of these ester-type gum bases and to appropriately manage their quality control, it is necessary to establish their specifications.

Specifications for some ester-type gum bases such as beeswax, candelilla wax, and carnauba wax have been adopted by the Joint FAO/WHO Expert Committee on Food Additives (JECFA; FAO: Food and Agriculture Organization of the United Nations; WHO: World Health Organization), the EU, and the USA. In Japan, the use of many varieties of ester-type gum bases as natural food additives is allowed. However, for the most ester-type gum bases, there is no specification in the Japanese food additive regulation. To establish official specifications for these ester-type gum bases, a simple analytical method for confirmation and discrimination of the different ester-type gum bases is required. We previously reported (Tada et al. 2007) an analytical method for 10 types of food additive gum bases such as lanolin, beeswax, and jojoba wax using gas chromatography/mass spectrometry (GC/MS) following hydrolysis and derivatization of the wax esters. Using the method, major constitutive fatty acids and alcohols of the food additive gum bases were identified, and it was clarified that each ester-type gum base has a characteristic composition of these constituent fatty acids and alcohols. However, this is a time-consuming and complicated procedure. In addition, it does not provide information on the composition of the wax ester species in the gum bases or the combination of fatty acids and fatty alcohols in each wax ester.

Several direct analytical methods for wax esters have been reported (Moldovan et al. 2002a; Regert et al. 2006; Stránskzý et al. 2006; Fitzgerald and Murphy 2007; Vrkoslav et al. 2010, 2011; Zhang et al. 2010). However, GC/MS methods using conventional temperatures (Stránskzý et al. 2006; Fitzgerald and Murphy 2007; Zhang et al. 2010) can be applied only for wax esters with carbon numbers of ∼40 and are not suitable for the detection of wax esters with carbon numbers of 50–60. Because the volatilities of long-chain wax esters are extremely low, they are not sufficiently detectable via GC using conventional column temperatures, which are typically less than 330°C. Although liquid chromatography/atmospheric-pressure chemical ionization-mass spectrometry (LC/APCI-MS) and LC/APCI-MS/MS methods (Vrkoslav et al. 2010, 2011) have been applied for the detection of wax esters with carbon numbers of 52 and 54 and are more suitable for the detection of unstable highly unsaturated wax esters (Vrkoslav et al. 2010), these methods take longer than 100 min for the detection of wax esters with carbon numbers greater than 52. Furthermore, some direct high-temperature GC/MS for the detection of wax esters have been reported (Moldovan et al. 2002a; Regert et al. 2006; Vrkoslav et al. 2010), but the target materials for these analyses were mainly wax ester standards or sculptures. To the best of our knowledge, development of a direct GC/MS method for the determination of many varieties of gum bases used as food additives has not yet been achieved.

Therefore, we developed a high-temperature GC/MS method for the discrimination and identification of ester-type gum bases used as food additives via direct detection of the wax esters. With this method, the wax esters in gum bases can be directly analyzed with simultaneous identification of the constituent fatty acids of each wax ester using the MS spectrum of each ester peak.

## Materials and Methods

### Samples

Samples of nine types of natural gum bases used as food additives (one sample each of lanolin [LA-1], beeswax [BE-1], jojoba wax [JO-1], candelilla wax [CA-1], shellac wax [SH-1], carnauba wax [CR-1], rice bran wax [RI-1], and Japan wax [JA-1] and two samples of urushi wax [UR-1 and UR-2]), along with montan wax (MO-1), which was listed as a type of food additive in Japan up to May 2011, were obtained through the Japan Food Additives Association. Additional experimental reagents corresponding to the above food additives were purchased as follows: l. Lanolin anhydrous (MP Biomedical, LLC, Santa Ana, CA) (LA-2), lanolin (Alfa Aesar Co. Ltd., Ward Hill, MA) (LA-3), beeswax (white pellets, Wako Pure Chemical Industries, Ltd., Osaka, Japan) (BE-2), beeswax (yellow pellets, Wako Pure Chemical Industries, Ltd.), beeswax (yellow, Nacalai Tesque, Inc., Kyoto, Japan) (BE-3), beeswax (white, Nacalai Tesque, Inc.) (BE-4), carnauba wax (Wako Pure Chemical Industries, Ltd.) (CR-2), carnauba wax yellow (Acros Organics [part of Thermo Fisher Scientific, Geel, Belgium]) (CR-3). Jojoba oil (JO-2), two candelilla wax samples (CA-2 and CA-3), rice bran wax (RI-2), Japan wax (for cosmetics) (JA-2), Japan wax (unbleached) (JA-3), and Japan wax (bleached) (JA-4) were purchased from Japanese online shops.

### Standards for GC/MS analysis

Fatty acid methyl ester standards: An oil reference standard (containing various types of fatty acid methyl esters) (Supelco, Bellefonte, PA), methyl 16-methylhepta-decanoate, methyl 12-hydroxystearate, and dl-*α*-hydroxystearic acid methyl ester were purchased from Sigma-Aldrich Co., St. Louis, MO. Methyl lignolenate (18:3) (Fluka, Buchs, Switzerland) was purchased from Wako Pure Chemical Industries Ltd. Fatty acid methyl esters (saturated straight chains) and a bacterial acid methyl ester CP mix were manufactured by Supelco.

Long-chain ester standards: Stearyl arachidate, arachidyl arachidate, behenyl arachidate, oleyl behenate, arachidyl behenate, behenyl behenate, behenyl oleate, and behenyl stearate manufactured by NU-CHEK-PREP Inc., Elysian, MN, were purchased from Funakoshi Co. Ltd., Tokyo, Japan. The esters, 2-octyldodecyl myristate, hexadecyl 2-ethylhexanoate, and isohexadecyl stearate were purchased from Wako Pure Chemical Industries, Ltd.

Glyceride standards: Tripalmitin (Fluka) was purchased from Sigma-Aldrich Co. Ltd. Glyceryl-1,2-palmitin-3-olein, glyceryl-1,3-palmitin-2-olein, glyceryl-1,2-palmitin-3-stearin, glyceryl-1,2-olein-3-palmitin, glyceryl-1-palmitin-2-olein-3-stearin, and glyceryl-1,2-stearin-3-palmitin were manufactured by Larodan AB Co. Ltd., Malmö, Sweden and were purchased along with 1,2-dipalmitin and 1-monopalmitin from Funakoshi Co. Ltd. All other chemicals were GC reagent grade and used without further purification.

### Instrumentation

The GC/MS system (Shimadzu Co. Ltd., Kyoto, Japan) consisted of a GC-17A gas chromatograph equipped with an MS-QP5050 mass spectrometer run in electron ionization (EI) mode using an AOC-20i auto injector.

### GC/MS analysis of wax esters in gum bases

GC/MS analysis of the wax esters was performed on a DB-1 HT fused-silica capillary column (15 m × 0.25 mm, film thickness of 0.10 *μ*m; J&W Scientific, Folsom, CA). The injector and detector temperatures were set at 390°C, and the column temperature was programed from 120°C to 240°C at 15°C/min and then from 240°C to 390°C at 8°C/min and finally maintained at 390°C for 6 min. Samples (1 *μ*L) were injected through a split-injector (1/5). MS spectra were detected in EI mode by scanning *m/z* values ranging 50–920. Samples and standards were dissolved in hexane, toluene, or ethanol (0.1–1.0 mg/mL). Each sample solution was injected in duplicate, and reproducibility of the results was confirmed.

## Results and Discussion

### Establishment of a direct analytical method for wax esters via high-temperature GC/MS on a capillary column

The major constituents of gum bases used as food additives are wax esters. They are composed of long-chain fatty acids and long-chain alcohols. Since the volatilities of these wax esters are very low, they are not sufficiently detected via GC using conventional column temperatures (<330°C). To overcome this problem, a GC/MS method was developed using a capillary column that enables high-temperature analysis for the direct detection of wax esters. The appropriate temperatures for the capillary column, injector, and detector were examined using standard wax ester mixtures. It was observed that, considering the sensitivity, the suitable temperature for the injector and detector was 390°C. In addition, an increasing program for the column temperature from 120°C to 390°C provided good separation of the structural isomers of the various triglycerides in the standard mixtures.

**Figure 1:**
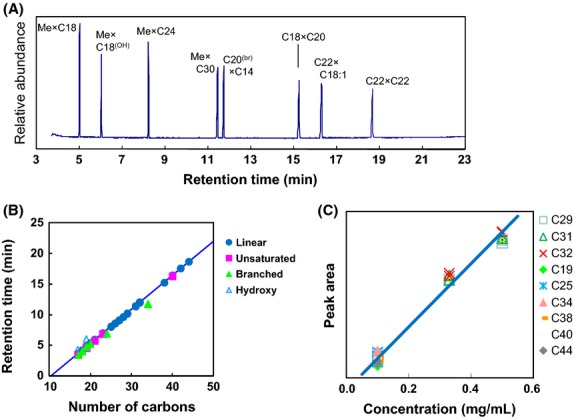
GC/MS analysis of long-chain esters standards. (A) Total ion chromatogram (TIC) of a standard mixture of esters. Esters are labeled as the carbon number of the constitutive alcohol × the carbon number of the constitutive fatty acid. Me, methanol; OH, hydroxy; br, branched; C18:1:C18 fatty acid with an unsaturated bond. (B) Correlation between the number of carbons in the standard esters and their retention times. Linear, unsaturated, branched, and hydroxy indicate the types of ester structures. (C) Correlation between the concentration of the standard esters and their peak areas in the TICs. The labels C29–C44 indicate esters with carbon numbers ranging from 29 to 44.

Figure 1A shows the GC/MS total ion chromatogram (TIC) of a standard mixture of various esters analyzed using the established method. This standard mixture contained straight-chain esters, branched-chain esters, saturated esters, unsaturated esters, and hydroxy esters with carbon numbers ranging from 19 to 44. All of the standard esters were well-separated within 19 min. As shown in Figure 1B, retention times of the standard esters approximately correlate with their carbon numbers, regardless of their structural type (Stránskzý et al. 2006; Zhang et al. 2010). These results suggest that the carbon number of esters can be estimated from the retention times of their peaks. Next, to assess the linearity of the relationship between peak areas in the TIC and the concentrations of the standard esters, calibration curves were constructed. As can be seen in Figure 1C, sufficient linearity for rough determination of the concentrations of wax esters with carbon numbers ranging from 29 to 44 is observed over the concentration range 0.1–0.5 mg/mL (correlation coefficient *R*^2^ = 0.9876). These results indicate that analysis of the TIC peak areas of the wax esters can be used to roughly determine the concentrations of the corresponding esters in the gum bases.

### Application of the established GC/MS method to the analysis of wax esters in food additive gum bases and experimental reagents

**Figure 2:**
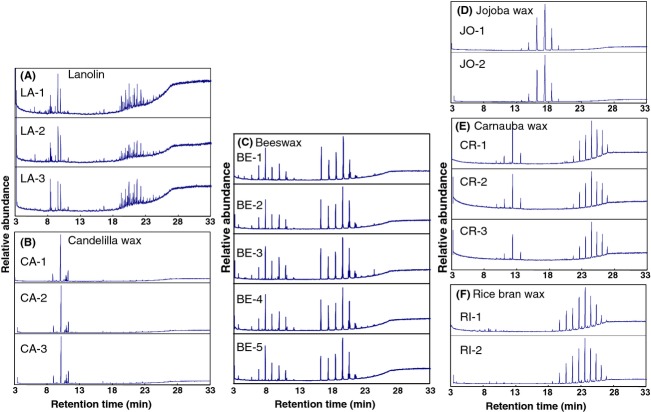
GC/MS TICs of food additive gum bases [LA-1 (A), CA-1 (B), BE-1 (C), JO-1 (D), CR-1 (E), and RI-1 (F)] and commercial samples.

To investigate the types and quantities of esters contained in various gum base samples, TIC patterns of food additive gum bases and experimental reagents were determined using the established GC/MS method. As shown in Figure 2, similar TIC patterns were observed for gum bases derived from the same waxes. It was also confirmed that MS spectra of the TIC peaks were consistent with those of TIC peaks observed at identical retention times for gum bases derived from the same types of wax (data not shown). These results confirm that the gum bases formulated with the same types of wax have specific and characteristic TIC patterns and ester compositions.

**Figure 3:**
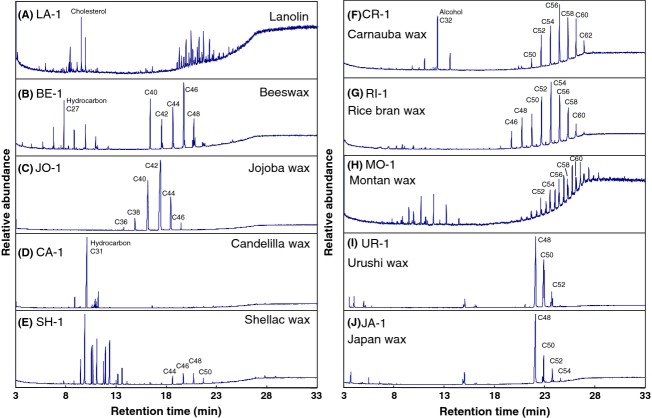
GC/MS TICs of 10 types of food additive gum bases. The number labels in the chromatograms indicate the number of carbons in the wax esters estimated from the retention times.

**Figure 4:**
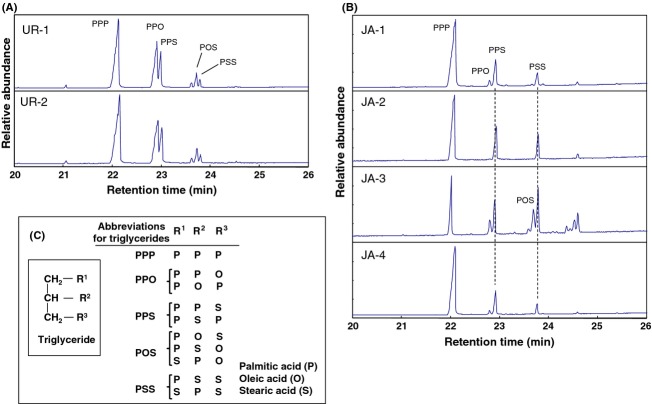
Magnified GC/MS TICs of (A) urushi waxes and (B) Japan waxes. (C) Abbreviations for the triglycerides are listed in the table (C). UR-1, UR-2, and JA-1 are food additive gum bases, while the other waxes are commercial samples.

Next, GC/MS TIC patterns of 10 types of food additive gum bases derived from different types of wax were obtained (Fig. 3). Based on the results for the molecular ions and correlation between the retention times and ester carbon numbers (Fig. 1B), it was possible to estimate the carbon numbers of the wax esters represented by the individual TIC peaks. In addition, the other constituents were also identified using previously reported data (Asano 1977; Lawrence et al. 1982; Tonogai et al. 1985; Tachibana et al. 1992; Jover et al. 2002; Moldovan et al. 2002b; Bonaduce and Colombini 2004; Jiménez et al. 2004; Jin et al. 2006) and by comparison to the analyses results described above for the standards and libraries of MS spectra such as NIST 147, NIST 27, and Wiley 7. As shown in Figure 3, each TIC of the 10 types of food additive gum bases has a characteristic pattern. In addition, it was observed that the composition of major esters in the food additive gum bases was nearly the same as those previously reported (Asano 1977; Lawrence et al. 1982; Tonogai et al. 1985; Tachibana et al. 1992; Jover et al. 2002; Moldovan et al. 2002b; Bonaduce and Colombini 2004; Jiménez et al. 2004; Jin et al. 2006). For lanolin (Fig. 3A), wax esters with carbon numbers ranging from 48 to 52 were detected along with free cholesterols and sterol esters (Jover et al. 2002; Moldovan et al. 2002b), while in the chromatogram of beeswax (Fig. 3B), wax esters with carbon numbers ranging from 40 to 48 were detected along with hydrocarbons such as C_27_H_56_ (Bonaduce and Colombini 2004; Jiménez et al. 2004). For candelilla wax (Fig. 3D), saturated hydrocarbons such as C_31_H_64_ were the major constituents in accordance with a previous report (Lawrence et al. 1982; Tonogai et al. 1985). The shellac wax (Fig. 3E) contained wax esters with carbon numbers ranging from 44 to 50 along with free alcohols, free fatty acids, and hydrocarbons (Lawrence et al. 1982). As shown in Figure 3F–H, carnauba wax, rice bran wax, and montan wax contained similar wax esters in terms of their carbon numbers. However, these results suggest that the three gum bases may be distinguished by evaluating the TICs for the peaks of other characteristic constituents such as the alcohol with carbon numbers 32 in carnauba wax (Lawrence et al. 1982; Tonogai et al. 1985) and hydrocarbons in montan wax (Asano 1977; Lawrence et al. 1982). As previously reported (Tachibana et al. 1992; Jin et al. 2006), major components of urushi and Japan waxes were found to be triglycerides (Fig. 3I and J). As can be seen in the magnified chromatograms of urushi and Japan waxes (Fig. 4A and B, respectively). The ratio of glycerol 1,2-dipalmitate 3-oleate (PPO) to glycerol tripalmitate (PPP) (table in Fig. 4C for abbreviation definitions) for urushi wax (Fig. 4A) is higher than that for the Japan wax (Fig. 4B) (Tachibana et al. 1992; Jin et al. 2006). Accordingly, these two gum bases can be distinguished using this ratio.

These results thus demonstrate that food additive gum bases can be distinguished from the other based on the TIC patterns obtained using the established direct GC/MS analysis (without hydrolysis and derivatization) of the esters in these food additives.

### Comparison of the MS chromatograms of carnauba wax, rice bran wax, and montan wax

**Figure 5:**
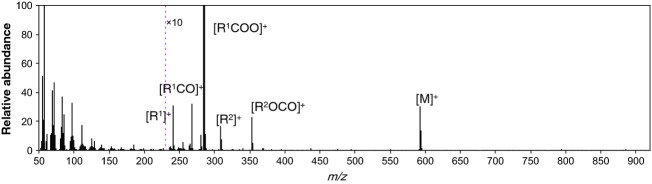
Mass spectra of C40 ester standards (C22:0 alcohol × C18:0 fatty acid, MW = 592) obtained using GC/MS method.

As shown in Figure 3F–H, it was difficult to differentiate carnauba wax, rice bran wax, and montan wax using the TICs alone. Therefore, the MS chromatograms were analyzed. As shown in Figure 5, the MS spectrum of a standard of the saturated straight-chain ester, behenyl stearate), obtained using the established GC/MS method contained product ions derived from the fatty acid moiety of the ester ([R^1^COO]^+^, [R^1^CO]^+^, and [R^1^]^+^) and product ions derived from the alcohol moiety ([R^2^]^+^ and [R^2^OCO]^+^). It was observed that the product ions corresponding to the fatty acid and alcohol moieties of standard wax esters are generally observed in their MS spectra under these conditions. These results suggest that analysis of the MS spectra obtained using the established method can provide information on the constitutive fatty acids and alcohols of the esters.

**Figure 6:**
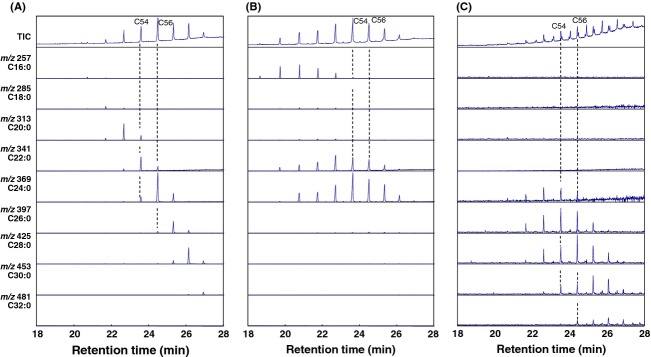
Mass chromatograms of the fragment ions derived from the constitutive fatty acids of the wax esters in (A) carnauba wax, (B) rice bran wax, and (C) montan wax via GC/MS analysis. For comparison, the TICs of the waxes are also shown (top of each figure). Number labels in the TICs indicate the carbon numbers of the wax esters.

Therefore, to more clearly distinguish between carnauba wax, rice bran wax, and montan wax, the MS chromatograms of the product ions derived from the constitutive fatty acids of the esters in these three waxes were compared. As can be seen in Figure 6, [RCOO]^+^ product ions, of the constitutive fatty acids of the esters in the three waxes observed in the MS chromatograms, included C16:0 (*m/z* = 257), C18:0 (*m/z* = 285), C20:0 (*m/z* = 313), C22:0 (*m/z* = 341), C24:0 (*m/z* = 369), C26:0 (*m/z* = 397), C28:0 (*m/z* = 425), C30:0 (*m/z* = 453), and C32:0 (*m/z* = 481). These product ions were reanalyzed considering the data obtained using the GC/MS method established in this study. As can be seen in the TICs of the three gum bases (Fig. 6), wax esters with carbon numbers ranging from 50 to 56 were detected at retention times of 21–27 min. In the MS chromatogram of carnauba wax (Fig. 6A), carbon numbers of the major constitutive fatty acids of the esters increased with an increase in the carbon numbers of their corresponding esters. On the other hand, the esters detected in rice bran wax consisted of only three fatty acids C16:0, C22:0, and C24:0, regardless of the carbon number of the esters (Fig. 6B). In addition, as can be seen in Figure 6C, the esters detected in montan wax consisted of fatty acids with longer chains (C24:0–C32:0) than those of the esters detected in the other two gum bases. On the basis of these results, compositions of the constitutive fatty acids in the C54 and C56 esters in the three gum bases were then compared (Table 1). As shown in Table 1, among the three gum bases, composition of the constitutive fatty acids in the esters with identical carbon numbers were clearly different. These results indicate that these three gum bases can be distinguished by comparing the mass chromatograms and TICs obtained using the newly developed direct GC/MS method.

**Table 1:** Estimated composition (%) of the constitutive fatty acids in the C54 and C56 wax esters detected in carnauba wax, rice bran wax, and montan wax based on the mass chromatograms of their fragment ions

	Compositions (%)					
	
	C54 Wax ester			C56 Wax ester		
		
Constitutive fatty acids	Carnauba wax	Rice bran wax	Montan wax	Carnauba wax	Rice bran wax	Montan wax
C16:0	–	1	–	–	–	–
C18:0	–	1	–	–	–	–
C20:0	17	1	–	–	0	–
C22:0	61	28	–	11	29	–
C24:0	22	69	19	84	70	10
C26:0	–	–	39	5	–	29
C28:0	–	–	29	–	–	40
C30:0	–	–	13	–	–	15
C32:0	–	–	–	–	–	6

–: Not detected.

We previously reported (Tada et al. 2007) an analytical method for the determination of food additive gum bases using GC/MS after hydrolysis and derivatization, and identified the major constitutive fatty acids and alcohols of the gum bases. However, the method does not provide information on the constitutive fatty acid for each respective ester in the gum bases. With the present analytical method, the esters in the gum bases can be directly analyzed with simultaneous prediction of the constitutive fatty acids of the corresponding esters using the MS spectra of the ester peaks. In addition, this direct GC/MS method is simple, clear, and particularly useful for the rapid analysis of various types of food additive gum bases without the need for hydrolysis and derivatization of the esters in the gum bases.
